# Identification of microRNAs as potential biomarkers for lung adenocarcinoma using integrating genomics analysis

**DOI:** 10.18632/oncotarget.19358

**Published:** 2017-07-18

**Authors:** Zhuo Peng, Longfei Pan, Zequn Niu, Wei Li, Xiaoyan Dang, Lin Wan, Rui Zhang, Shuanying Yang

**Affiliations:** ^1^ Department of Emergency Medicine, Second Affiliated Hospital, Xi'an Jiaotong University, Xi'an 710004, Shaanxi Province, China; ^2^ Department of Respiratory Medicine, Second Affiliated Hospital, Xi'an Jiaotong University, Xi'an 710004, Shaanxi Province, China

**Keywords:** microRNAs, lung adenocarcinoma, robust rank aggregation

## Abstract

Lung adenocarcinoma (LUAD) is the most common histological subtype of non-small cell lung cancer, but novel biomarkers for early diagnosis are lacking. Extensive effort has been exerted to identify miRNA biomarkers in LUAD. Unfortunately, high inter-lab variability and small sample sizes have produced inconsistent conclusions in this field. To resolve the above-mentioned limitations, we performed a comprehensive analysis based on LUAD miRNome profiling studies using the robust rank aggregation (RRA) method. Moreover, miRNA-gene interaction network, pathway enrichment analysis and Kaplan-Meier survival curves were used to investigate the clinical values and biological functions of the identified miRNAs. A total of six common differentially expressed miRNAs (DEMs) were identified in LUAD. An independent cohort further confirmed that four miRNAs (miR-21-5p, miR-210-3p, miR-182-5p and miR-183-5p) were up-regulated and two miRNAs (miR-126-3p and miR-218-5p) were down-regulated in LUAD tissues. Pathway enrichment analysis also suggested that the above-listed six DEMs may affect LUAD progression via the estrogen signaling pathway. Survival analysis based on the TCGA dataset revealed the potential prognostic values of six DEMs in patients with LUAD (*P*-value<0.01). In conclusion, we identified a panel of six miRNAs from LUAD using miRNome profiling studies. Our results provide evidence for the use of these six DEMs as novel diagnostic and prognostic biomarkers for LUAD patients.

## INTRODUCTION

The incidence and mortality of lung cancer have increased steadily worldwide in the past several decades. Lung cancer is a fatal disease that is tightly associated with cigarette smoking. Apart from other histological subtypes, lung adenocarcinoma (LUAD) is the most common histological subtype among never-smokers [1]. Despite many achievements made in anti-cancer therapy over years, the survival of LUAD is far from satisfactory. Due to a lack of specific clinical symptoms, most patients with LUAD are diagnosed at a late stage, leaving little chance for effective treatment. Thus, exploring novel cancer-specific biomarkers for LUAD patients will help monitor tumor progression and guide clinical treatment.

MiRNAs are a group of small molecules that facilitate tumor progression via regulation of the function of protein-coding mRNAs [2]. MiRNAs are stable molecules that exist in tissue and fluid samples and exhibit potential as biomarkers for diagnostic, prognostic and therapeutic applications in multiple malignancies [3]. The application of high-throughput miRNA profiling methods during the past decade, such as RNA sequencing and microarrays, has enabled researchers to identify a group of miRNAs as biomarkers in cancer diagnosis [4]. The majority of previous studies were primarily based on small specimen sizes and various technological platforms, and no consistent conclusion has ever been made. To overcome these limitations, Vosa et al developed a robust rank aggregation (RRA) method for comparing several ranked gene lists and identifying the most commonly overlapping miRNAs [5]. By applying the RRA method, Vosa et al identified 15 aberrantly expressed miRNAs in lung cancer [6]. However, there has been no attempt to investigate LUAD-specific miRNAs using the RRA method.

In the present study, we first performed RRA analysis based on high-throughput miRNome profiling studies and identified candidate miRNAs in LUAD tissues. We next validated the aberrant miRNAs identified in the first step in an independent cohort. Finally, we predicted the biological functions of the above-identified DEMs and investigated the clinical values of these miRNAs in The Cancer Genome Atlas (TCGA) cohort.

## RESULTS

### Characteristics of included miRNome profiling studies

A total of nine miRNome profiling studies retrieved from the PubMed database (https://www.ncbi.nlm.nih.gov/pubmed/) were used to construct the lung adenocarcinoma miRNA expression profiling datasets [7–15]. Supplementary Table 1 lists the basic characteristics of the included nine miRNome profiling studies, including the first author, publication year, first author initials, country, population ethnicity, sample number (tumor tissues and normal/benign tissues), sample source (frozen or formalin-fixed paraffin-embedded tissues), detection methods, detected miRNA number, and cut-off criteria for miRNA selection. The pooled dataset included 595 cancer and 168 non-cancerous tissue samples. Various microarray platforms were used in the studies, such as high-throughput quantitative PCR (qPCR) and microarray-based and second-generation sequencing methods. The numbers of detected miRNAs in each study varied from 84 to 1046.

### Differentially expressed miRNAs (DEMs) in LUAD

**Figure 1:**
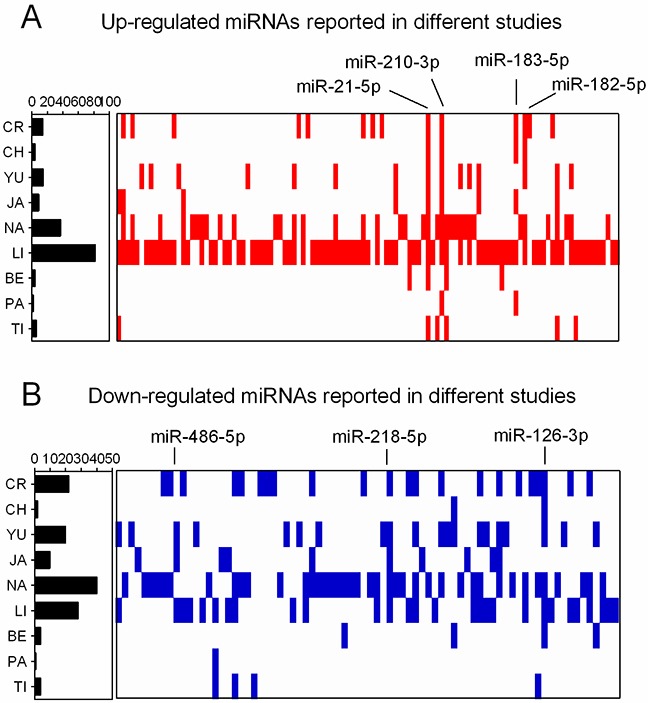
Distribution of DEMs in miRNome profiling studies **(A)** Up-regulated miRNAs reported in nine LUAD miRNome profiling studies. **(B)** Down-regulated miRNAs reported in nine LUAD miRNome profiling studies. The positions of DEMs were assigned in the figure.

**Figure 2:**
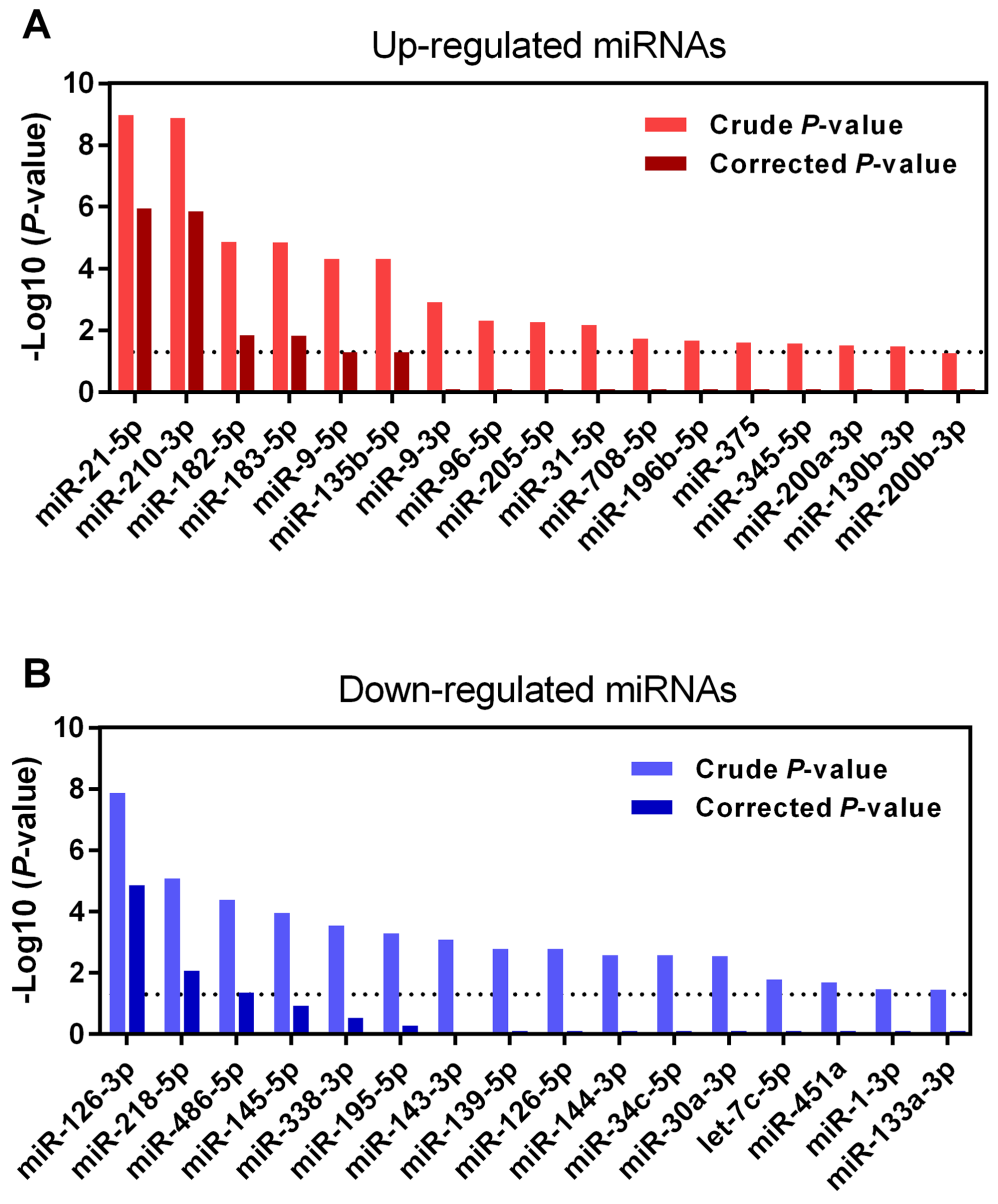
Ranks of six DEMs in miRNome profiling studies **(A)** The ranks of four up-regulated miRNAs (miR-21-5p, miR-210-3p, miR-182-5p and miR-183-5p) in nine LUAD miRNome profiling studies. **(B)** The ranks of two down-regulated miRNAs (miR-126-3p and miR-218-5p) in nine LUAD miRNome profiling studies.

**Figure 3:**
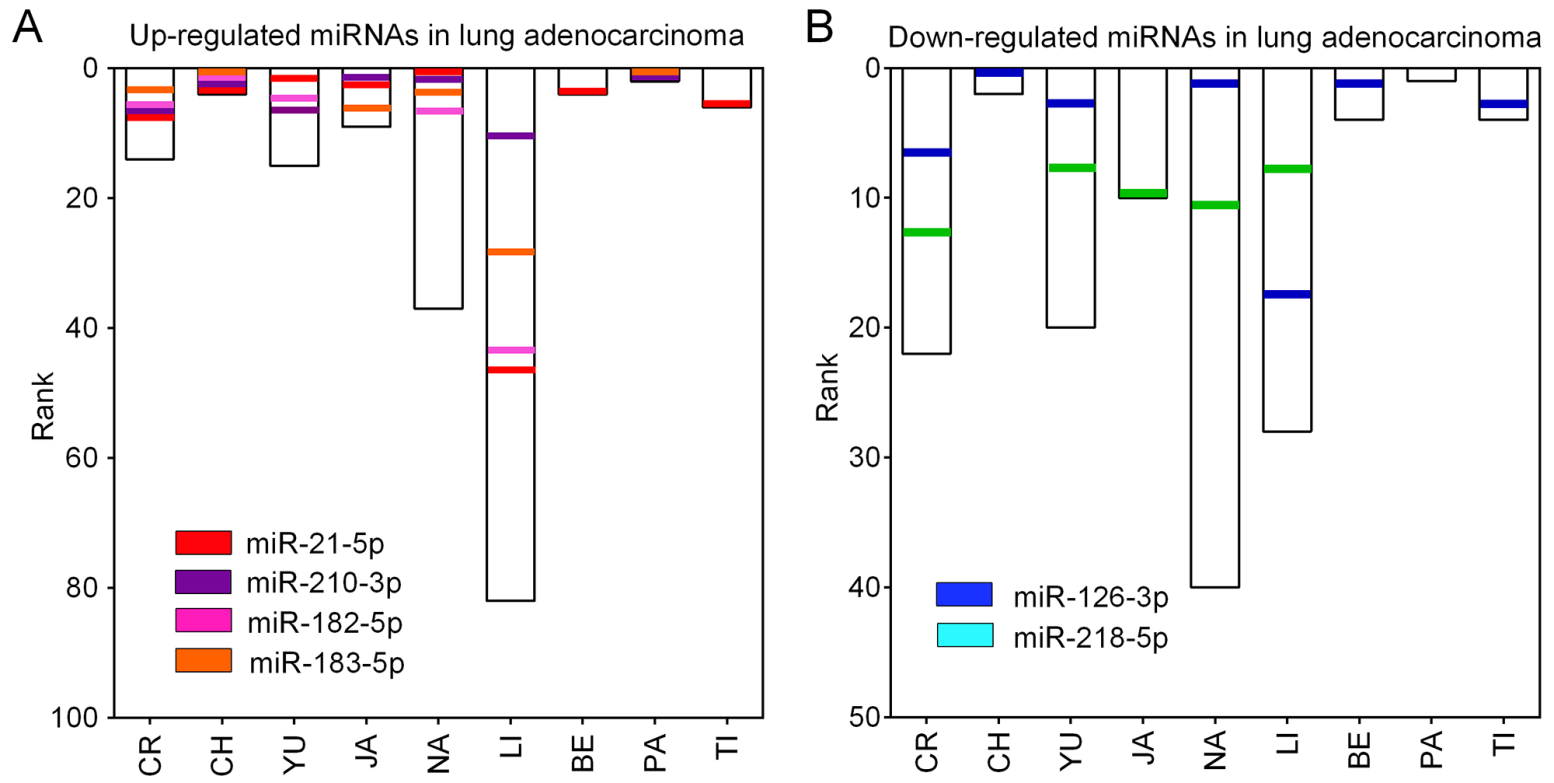
RRA analysis of DEMs in LUAD **(A)** Up-regulated and **(B)** down-regulated miRNAs in LUAD were re-ranked according to their crude and Bonferroni-corrected *P*-values. Only six miRNAs (four up-regulated: miR-21-5p, miR-210-3p, miR-182-5p and miR-183-5p; two down-regulated: miR-126-3p and miR-218-5p) reached statistical significance with Bonferroni-corrected *P*-values less than 0.05 (−Log10 [*P*-value]>1.30).

Figure 1 shows the 109 up-regulated and 78 down-regulated miRNAs that were reported in at least one study. Eighteen up-regulated (16.5%, 18/109) and 13 down-regulated (16.7%, 13/78) miRNAs were reported in more than three studies. As previously reported [16], we performed an integrated analysis using the R package “Robust Rank Aggreg”. We primarily identified a statistically significant panel of 16 up-regulated miRNAs (miR-21-5p, miR-210-3p, miR-182-5p, miR-183-5p, miR-9-5p, miR-135b-5p, miR-9-3p, miR-96-5p, miR-205-5p, miR-31-5p, miR-708-5p, miR-196b-5p, miR-375, miR-345-5p, miR-200a-3p, and miR-130b-3p) and 16 down-regulated miRNAs (miR-126-3p, miR-218-5p, miR-486-5p, miR-145-5p, miR-338-3p, miR-195-5p, miR-143-3p, miR-139-5p, miR-126-5p, miR-144-3p, miR-34c-5p, miR-30a-3p, let-7c-5p, miR-451a, miR-1-3p, and miR-133a-3p) (Table 1). The direction of expression change of these miRNAs was consistent across all miRome profiling studies, with *P*-values ranging from 3.47E-02 to 1.09E-09. Four of the up-regulated miRNAs (miR-21-5p, miR-210-3p, miR-182-5p and miR-183-5p) and two down-regulated miRNAs (miR-126-3p and miR-218-5p) remained statistically significant after Bonferroni correction (corrected *P*-value from 1.49E-02 to 1.14E-06) (Table 1 and Figure 2). These six miRNAs exhibited relatively high rank scores across the included studies, which supports their potential clinical values as biomarkers for LUAD (Figure 3).

**Table 1:** Differentially expressed miRNAs in lung adenocarcinoma

microRNA	Chromosome	*P*-value	Corrected *P*-value
***Up-regulated***			
miR-21-5p	17q23.1	1.09E-09	1.14E-06
miR-210-3p	11p15.5	1.35E-09	1.41E-06
miR-182-5p	7q32.2	1.38E-05	1.44E-02
miR-183-5p	7q32.2	1.42E-05	1.49E-02
miR-9-5p	1q22	4.84E-05	5.07E-02
miR-135b-5p	1q32.1	4.92E-05	5.15E-02
miR-9-3p	5q14.3	1.22E-03	1.00E+00
miR-96-5p	7q32.2	4.85E-03	1.00E+00
miR-205-5p	1q32.2	5.39E-03	1.00E+00
miR-31-5p	9p21.3	6.56E-03	1.00E+00
miR-708-5p	11q14.1	1.88E-02	1.00E+00
miR-196b-5p	7p15.2	2.11E-02	1.00E+00
miR-375	2q35	2.43E-02	1.00E+00
miR-345-5p	14q32.2	2.64E-02	1.00E+00
miR-200a-3p	1p36.33	3.07E-02	1.00E+00
miR-130b-3p	22q11.21	3.31E-02	1.00E+00
***Down-regulated***			
miR-126-3p	9q34.3	1.34E-08	1.40E-05
miR-218-5p	4p15.31	8.33E-06	8.71E-03
miR-486-5p	8p11.21	4.16E-05	4.35E-02
miR-145-5p	5q32	1.10E-04	1.15E-01
miR-338-3p	17q25.3	2.87E-04	3.00E-01
miR-195-5p	17p13.1	5.04E-04	5.28E-01
miR-143-3p	5q32	8.14E-04	8.51E-01
miR-139-5p	11q13.4	1.66E-03	1.00E+00
miR-126-5p	9q34.3	1.66E-03	1.00E+00
miR-144-3p	17q11.2	2.63E-03	1.00E+00
miR-34c-5p	11q23.1	2.69E-03	1.00E+00
miR-30a-3p	6q13	2.88E-03	1.00E+00
let-7c-5p	21q21.1	1.65E-02	1.00E+00
miR-451a	17q11.2	2.00E-02	1.00E+00
miR-1-3p	18q11.2	3.39E-02	1.00E+00
miR-133a-3p	20q13.33	3.47E-02	1.00E+00

### Validation of DEMs in LUAD tissues

**Figure 4:**
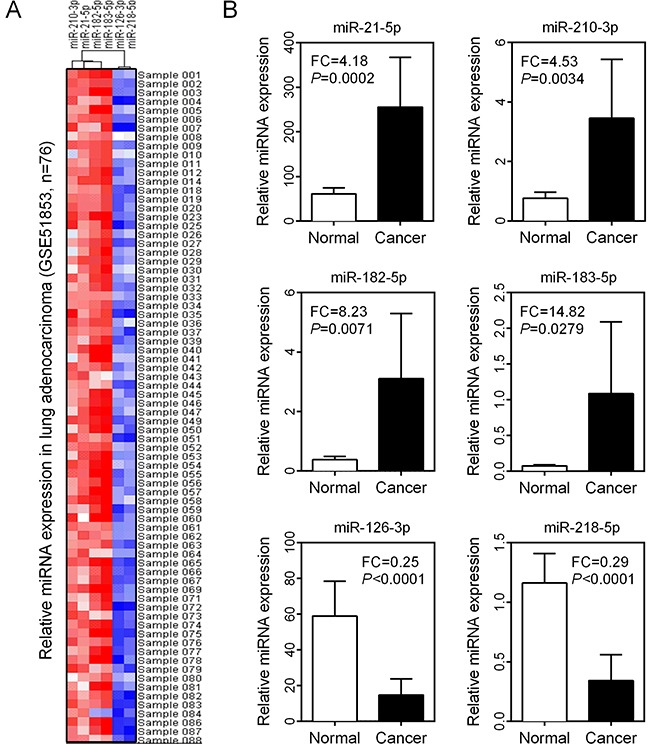
Expression validation of six DEMs in LUAD **(A)** Expression levels of six DEMs in 76 LUAD tissue samples were visualized as a heatmap. **(B)** Comparison of six DEM expression levels between normal and cancer groups.

To further validate the expression levels of six DEMs in LUAD patients, we downloaded an independent cohort (GSE51853) from the GEO database (https://www.ncbi.nlm.nih.gov/geo/), which contained five different histological types of lung cancer, including adenocarcinoma (n=76), adenosquamous carcinoma (n=4), large cell carcinoma (n=15), large cell neuroendocrine carcinoma (n=2) and squamous cell carcinoma (n=29). In this microarray dataset, normal lung tissues were used as controls in this microarray dataset. MiRNA expression profiles in LUAD patients revealed that miR-21-5p, miR-210-3p, miR-182-5p and miR-183-5p were overexpressed in nearly all samples. In contrast, miR-126-3p and miR-218-5p were significantly under-expressed (Figure 4A). Comparison of miRNA expression patterns between LUAD and normal lung tissues also revealed that miR-21-5p, miR-210-3p, miR-182-5p and miR-183-5p were significantly up-regulated and miR-126-3p and miR-218-5p were down-regulated in LUAD tissues (all *P*<0.01, Figure 4B).

### Functional prediction of DEMs

**Figure 5:**
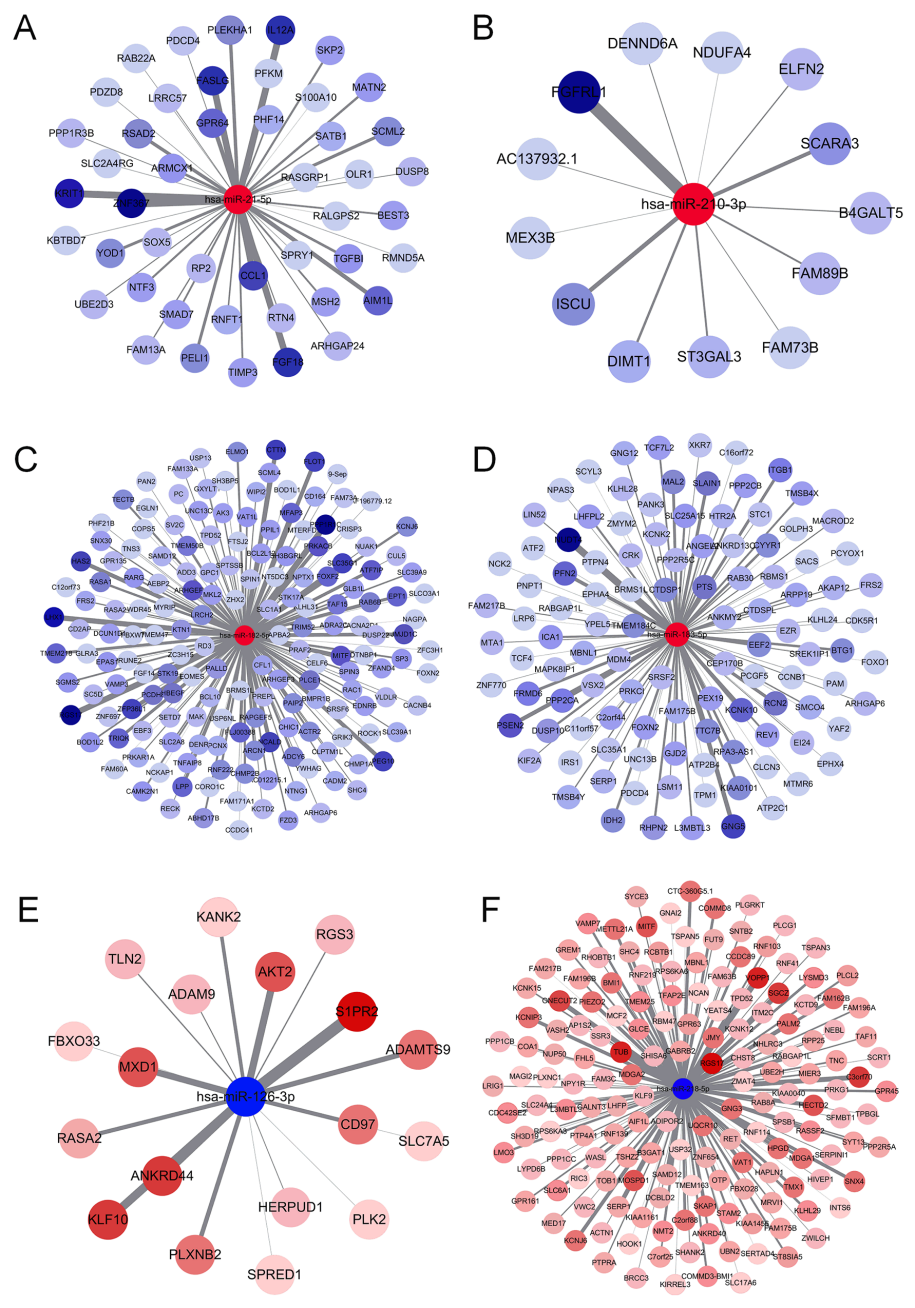
MiRNA-gene interaction network **(A)** miR-21-5p, **(B)** miR-210-3p, **(C)** miR-182-5p, **(D)** miR-183-5p, **(E)** miR-126-3p and **(F)** miR-218-5p.

**Figure 6:**
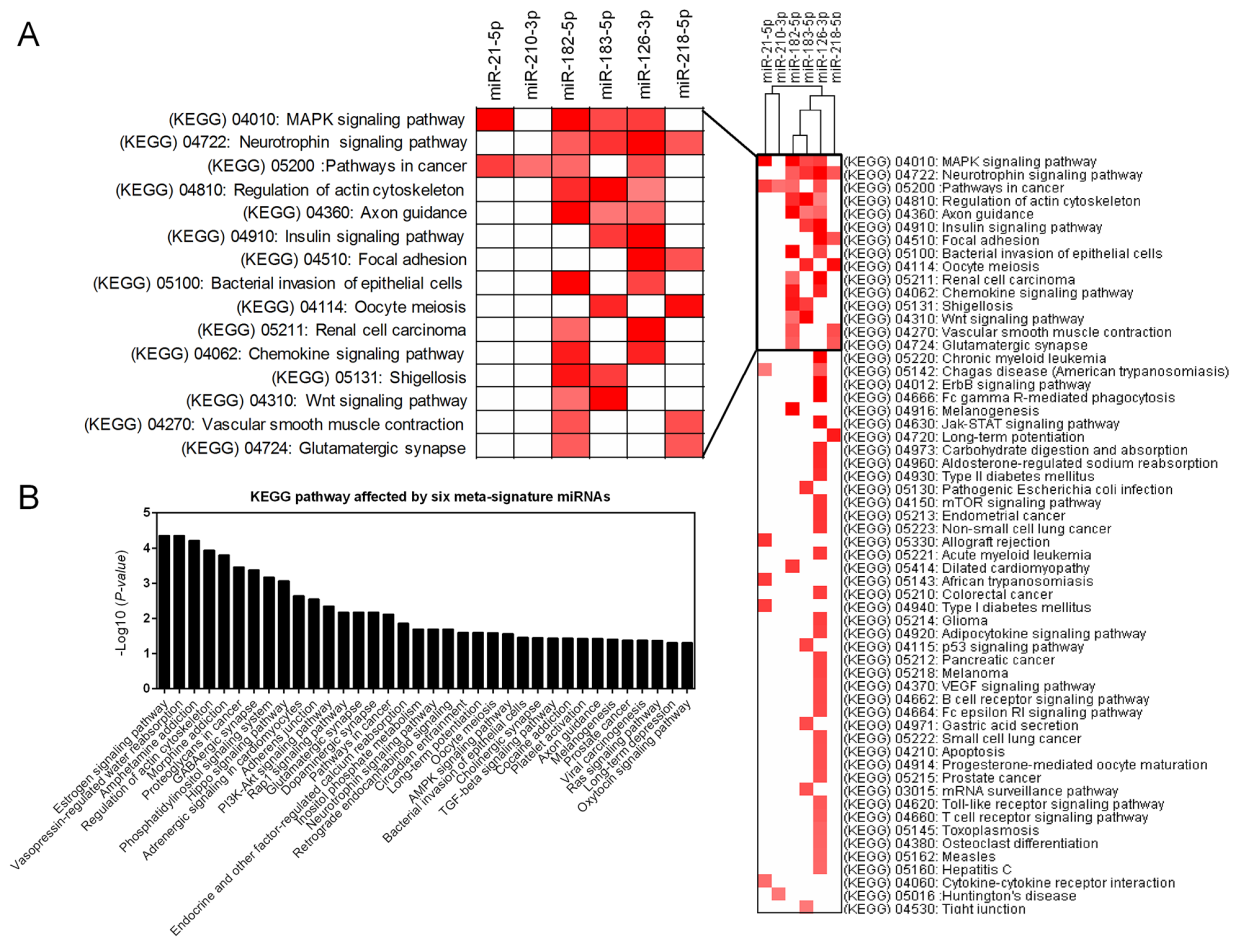
Pathway enrichment analysis of predicted targets of DEMs **(A)** Heatmap of the KEGG pathway enrichment of the target genes of six DEMs. Rows: pathways; Columns: genes. Range of colors (deep red to white) represented the –log10 (FDR). **(B)** The combinatorial effects of six DEMs in KEGG pathways.

The main function of miRNA is the silencing of gene expression via binding to specific target sites. Therefore, to elucidate the biological function of DEMs, we performed miRNA-target interaction and pathway enrichment of predicted targets. All predicted targets of each miRNA were obtained from TargetScan (http://www.targetscan.org/vert_71/), and only those targets with a cumulative weighted context score less than −0.4 were selected for further analysis. Figure 5 shows the miRNA-target interaction of each candidate miRNA using Cytoscape software (version 3.4.0). The number of predicted targets of a single miRNA ranged from 13 to 169. Seventeen genes (*ARHGAP6*, *BRMS1L*, *FAM175B*, *FAM217B*, *FRS2*, *KCNJ6*, *L3MBTL3*, *MBNL1*, *MITF*, *PDCD4*, *RABGAP1L*, *RASA2*, *RGS17*, *SAMD12*, *SERP1*, *SHC4* and *TPD52*) were targeted by more than one miRNA. We also performed pathway enrichment analysis of the predicted targets. Figure 6A shows that five KEGG pathways, including the “MAPK signaling pathway”, “Neurotrophin signaling pathway”, “Pathway in cancer”, “Regulation of actin cytoskeleton” and “Axon guidance pathway”, were associated with more than three miRNAs. Another algorithm (DIANA-miRPath v3.0) predicted the pathways affected by multiple miRNAs as “Pathway in cancer”, “Regulation of actin cytoskeleton”, “MAPK signaling pathway” and “Axon guidance pathway”, which were also common pathways affected by the largest number of miRNAs (Table 2). Notably, we found that the “Estrogen signaling pathway” was the most significant pathway affected by six miRNAs (Figure 6B). Activation of the estrogen signaling pathway was a reported promoter for LUAD [17], and our findings support critical roles for these six miRNAs in LUAD development.

**Table 2:** The combinatorial effect of six DEMs in KEGG pathways

#	KEGG pathway	#miRNAs	#genes
1	Pathways in cancer	6	68
2	PI3K-Akt signaling pathway	5	59
3	Regulation of actin cytoskeleton	5	47
4	Proteoglycans in cancer	5	41
5	AMPK signaling pathway	5	25
6	Axon guidance	5	24
7	Prostate cancer	5	19
8	ECM-receptor interaction	5	15
9	Rap1 signaling pathway	4	40
10	Ras signaling pathway	4	34
11	Dopaminergic synapse	4	29
12	Oxytocin signaling pathway	4	29
13	Adrenergic signaling in cardiomyocytes	4	28
14	Viral carcinogenesis	4	28
15	Hippo signaling pathway	4	25
16	Neurotrophin signaling pathway	4	25
17	Oocyte meiosis	4	24
18	Cholinergic synapse	4	24
19	Platelet activation	4	24
20	Glutamatergic synapse	4	22
21	Retrograde endocannabinoid signaling	4	21
22	Estrogen signaling pathway	4	20
23	Morphine addiction	4	18
24	Adherens junction	4	18
25	GABAergic synapse	4	17
26	Amphetamine addiction	4	16
27	Long-term potentiation	4	16
28	Bacterial invasion of epithelial cells	4	16
29	Vasopressin-regulated water reabsorption	4	15
30	Cocaine addiction	4	11
31	Circadian entrainment	3	18
32	Melanogenesis	3	18
33	Phosphatidylinositol signaling system	3	17
34	Inositol phosphate metabolism	3	14
35	TGF-beta signaling pathway	3	14
36	Long-term depression	3	13
37	Endocrine and other factor-regulated calcium reabsorption	3	10

### The association between DEMs and clinical outcome in LUAD

**Figure 7:**
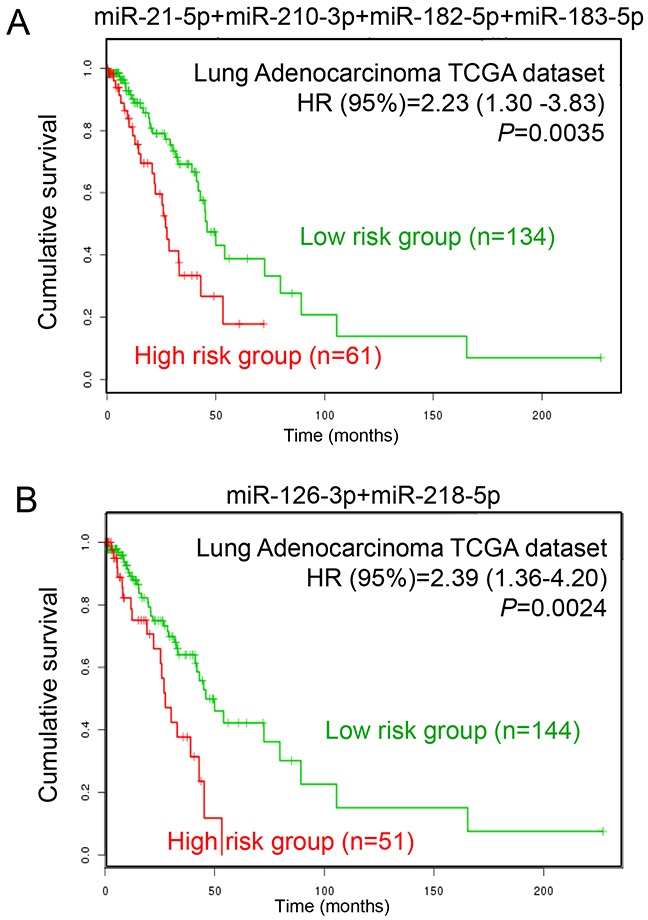
Prognostic analysis of LUAD patients with different miRNA expression levels **(A)** The prognostic values of four up-regulated miRNAs (miR-21-5p, miR-210-3p, miR-182-5p and miR-183-5p) in LUAD patients. **(B)** The prognostic values of two down-regulated miRNAs (miR-126-3p and miR-218-5p) in LUAD patients.

To explore the prognostic values of DEMs in LUAD patients, we obtained the LUAD TCGA dataset from SurvMicro (http://bioinformatica.mty.itesm.mx/SurvMicro), which is a web-based tool for assessing miRNA-based prognostic signatures [18]. We evaluated the association between miRNAs and patients’ overall survival using two panels, including four up-regulated miRNAs (miR-21-5p, miR-210-3p, miR-182-5p and miR-183-5p) and two down-regulated miRNAs (miR-126-3p and miR-218-5p). Patients in each panel were divided into two groups, high-risk and low-risk groups, according to the prognostic index calculated by multivariate survival analysis using the SurvMicro web tool. Figure 7A shows that the panel of four up-regulated miRNAs was significantly associated with overall survival of LUAD, with a *P*-value of 0.0035. Patients in the high-risk group exhibited lower cumulative survival rates than the low-risk group (hazard ratio [HR]=2.23, corresponding 95% confidence interval [95% CI]=1.30-3.83). Similarly, the panel of two down-regulated miRNAs was also significantly associated with overall survival in patients with LUAD (HR [95%CI]=2.39 [1.36-4.20], *P*=0.0024). These findings support the potential prognostic values of these six DEMs in survival prediction for LUAD patients.

## DISCUSSION

In the present study, we combined nine miRome profiling studies and identified LUAD-specific miRNAs from a total of 595 LUAD and 168 non-cancerous tissue samples using the RRA method. A panel of four up-regulated miRNAs (miR-21-5p, miR-210-3p, miR-182-5p and miR-183-5p) and two down-regulated miRNAs (miR-126-3p and miR-218-5p) were identified as commonly aberrantly expressed miRNAs in LUAD. Functional analysis revealed that these six miRNAs may be involved in LUAD development via modulation of the estrogen signaling pathway. Our clinical investigation further supports the prognostic value of these six miRNAs in LUAD patients.

In recent years, high-throughput profiling methods have been widely used to identify tumor-specific miRNAs as biomarkers for cancer diagnostic, prognostic and therapeutic applications [19, 20]. However, due to the use of different methods and platforms, the conclusions varied among those profiling studies. The RRA method was specifically designed to identify the most commonly overlapping factors and make these studies comparable [5]. An increasing number of studies have attempted to examine tumor specific-miRNAs using the RRA method. Researchers used this method in multiple malignancies, including pancreatic, liver, renal, breast, colon, ovarian, bladder, and nasopharyngeal tract cancer [16, 21–27]. Võsa U et al [6] first identified a panel of 15 aberrantly expressed miRNAs in lung cancer, including seven up-regulated miRNAs (miR-21, miR-210, miR-182, miR-31, miR-200b, miR-205 and miR-183) and eight down-regulated miRNAs (miR-126-3p, miR-30a, miR-30d, miR-486-5p, miR-451a, miR-126-5p, miR-143 and miR-145). Their results provided solid evidence for the use of these miRNAs as promising diagnostic and prognostic biomarkers in lung cancer. However, the authors mixed different histological types of lung cancer together and did not analyze miRNA signatures separately. Therefore, their findings based on mixed samples may have limited value in the clinic. Therefore, this study only included miRNome profiling studies of LUAD. Our findings improve our understanding of the molecular mechanism and identify more specific tumor biomarkers for LUAD [28].

Several of the miRNAs identified in our study, such as miR-21-5p and miR-210-3p, are functionally important in the progression of LUAD [29, 30]. When comparing our results with Võsa's results, we identified a specific down-regulated miRNA, miR-218-5p. Previous studies revealed that miR-218 acts as a tumor suppressor in many malignancies, such as glioma, head and neck squamous cell carcinoma, and cervical, nasopharyngeal, gastric and colon cancers [31–35]. In non-small cell lung cancer, miR-218 was reported to be significantly down-regulated and affect cell migration and invasion [36, 37]. Patients with low miR-218 expression exhibited a worse prognosis than patients with high miR-218 expression [38]. Using LUAD cell lines, Sher YP et al [39] found that the expression of miR-218 was significantly inhibited and involved in the process of brain metastasis. In our study, using an independent cohort, we also found that miR-218 was significantly down-regulated in LUAD tissues (fold change=0.29; *P*<0.0001). These reports support the need for further experimental and clinical exploration of miR-218 and other miRNAs in LUAD.

To further explore the underlying molecular association between DEMs and LUAD, in this study, we also performed miRNA-target interaction network and pathway enrichment analyses. We found that several KEGG pathways, such as “Regulation of actin cytoskeleton” and “MAPK signaling pathway”, were affected by nearly all six miRNAs. A very recent study demonstrated that “Regulation of actin cytoskeleton” and “MAPK signaling pathway” contribute to disease progression and drug resistance in LUAD cells [40], which suggests the critical role of DEMs in LUAD. Notably, we also found that the “Estrogen signaling pathway” was the most significant pathway affected by the six miRNAs, with a *P*-value of 4.44E-05. For decades, epidemiological studies have demonstrated an increase in LUAD in women. A mouse model demonstrated that estrogen is an essential factor during LUAD carcinogenesis [17]. Ikeda K et al [41] observed that the estrogen concentration in LUAD was significantly higher than controls. Moreover, a clinical investigation revealed that postmenopausal women who received estrogen plus progestin treatment exhibit an increased number of deaths from lung cancer [42]. The estrogen signaling pathway was also involved in cellular cytoskeleton organization [43] and cross-talk with the MAPK signaling pathway [44]. Taken together, biological function analyses of these six miRNAs strongly suggest critical roles in LUAD, which may provide a new strategy for LUAD diagnosis and prognosis.

In conclusion, we identified a panel of six miRNAs (miR-21-5p, miR-210-3p, miR-182-5p and miR-183-5p, miR-126-3p and miR-218-5p) in LUAD and evaluated their expression and prognostic values in an independent cohort. Our biological informatics analysis provided evidence for the important roles of these miRNAs in LUAD via modulation of the estrogen signaling pathway. Our findings warrant further clinical investigations to validate these six miRNAs as biomarkers for the early diagnosis and prognosis prediction of LUAD.

## MATERIALS AND METHODS

### Selection strategy for miRNome profiling studies

MiRNome profiling studies that used high-throughput methods, including second-generation sequencing, miRNA microarray and polymerase chain reaction (PCR) methods, which were designed for the parallel quantification of large numbers of miRNAs (96- or 384-well microplates) in LUAD tissue were included. MiRNome profiling studies based on only cell lines and non-LUAD subtypes were excluded. Rank lists of aberrantly expressed miRNAs from each study were mapped according to miRBase Version 21. Non-human miRNA, including viral and other species’ miRNAs, were excluded. The miRNA lists were separately recorded as up- or down-regulated miRNAs.

### Robust rank aggregation analysis

RRA analysis was performed using R software and an R package named “Robust Rank Aggreg” (freely available at comprehensive R Archive Network website, http://cran.r-project.org/). All miRNA lists were re-ranked according to the *P*-value assigned for each miRNA. Bonferroni corrections of *P*-values were calculated to avoid false positive results. Only corrected *P*-values less than 0.05 were considered statistically significant.

### MiRNA-gene interaction network analysis

The TargetScan web tool (www.targetscan.org/) was used to predict the gene target of each DEM. Only gene targets with a cumulative weighted context score<-0.4 were selected for inclusion in the miRNA-gene interaction network. The miRNA-gene interaction network was visualized using Cytoscape software (version 3.4.0).

### KEGG pathway enrichment analysis

KEGG pathway enrichment analysis for the predicted gene targets of each DEM was performed using the DAVID web tool (https://david.ncifcrf.gov/). The false discovery rate (FDR) of each KEGG pathway was transformed by -log10 and visualized as a heatmap. The combinatorial effects of multiple miRNAs in KEGG pathways were calculated using the miRPath algorithm in the DIANA web tool (http://www.microrna.gr/miRPathv2).

### Survival analysis

LUAD patients from The Cancer Genome Atlas (TCGA) cohort were used to investigate the prognosis values of DEMs in LUAD. The prognostic data and Kaplan–Meier curve were calculated on SurvMicro web tools (http://bioinformatica.mty.itesm.mx/SurvMicro).

### Statistical analysis

All data in the present study were analyzed using GraphPad Prism 6.0 software. The expression of each miRNA is expressed as a mean ± standard error of measurement (SD). Comparisons of each miRNA between cancer and normal groups were performed using Student's *t*-test. A *P*-value less than 0.05 was considered statistically significant.

## SUPPLEMENTARY MATERIALS TABLE


