# Supplementary material for: Identification of microRNAs as potential biomarkers for lung adenocarcinoma using integrating genomics analysis
Source: Oncotarget. 2017 Jul 18;8(38):64143–56. doi: 10.18632/oncotarget.19358 (PMC5609990; doi:10.18632/oncotarget.19358)
Supplement: Supplementary file 1 [file oncotarget-08-64143-s001.pdf]

Identification of microRNAs as potential biomarkers for lung adenocarcinoma using integrating genomics analysis

SUPPLEMENTARY MATERIALS

Supplementary Table 1: Characteristics of studies included for DEMS expression in LUAD

Author (year)	Acronym	Country	Ethnicity	No. of sample (T/N)	Sample source	Method	Total miRNAs	Cut-off criteria
Crawford (2009)	CR	USA	Caucasian	8/4	Frozen tissue	High-throughput qPCR	500	FDR<0.005
Cho (2009)	CH	China	Asian	10/10	Frozen tissue	Agilent Human miRNA Array	470	FC>2 and *P*<0.05
Yu (2010)	YU	USA	Caucasian	20/20	Frozen tissue	TaqMan-based miRNA array	377	FC>1.5 and *P*<0.01
Jane (2012)	JA	USA	Caucasian	56/56	56 frozen tissue and 47 FFPE	Illumina microarray V2	858	FC>1.5 and *P*<0.01
Nadal (2014)	NA	USA	Caucasian	91/10	Frozen tissue	TaqMan-based miRNA array	754	FDR<0.01
Li (2014)	LI	USA	Caucasian	385/46	Various	Illumina HiSeq Systems	1046	FC>2 and *P*<0.05
Begum (2015)	BE	USA	Caucasian	3/3	FFPE	Custom microarray	688	NA
Pak (2015)	PA	Korea	Asian	13/10	FFPE	PCR array	84	*P*<0.05
Tian (2016)	TI	China	Asian	9/9	Frozen tissue	RNA sequencing	256	FC>3

FFPE, formalin-fixed paraffin-embedded; FC, fold change; NA, not available.
